# Second Blood Meal by Female* Lutzomyia longipalpis*: Enhancement by Oviposition and Its Effects on Digestion, Longevity, and* Leishmania* Infection

**DOI:** 10.1155/2018/2472508

**Published:** 2018-03-25

**Authors:** C. S. Moraes, K. Aguiar-Martins, S. G. Costa, P. A. Bates, R. J. Dillon, F. A. Genta

**Affiliations:** ^1^Laboratory of Insect Biochemistry and Physiology, Oswaldo Cruz Institute, FIOCRUZ, 4365 Brasil Av., Leonidas Deane Building, Room 207, 21040-360 Manguinhos, RJ, Brazil; ^2^Faculty of Health and Medicine, Division of Biomedical and Life Sciences, Lancaster University, Furness Building, Bailrigg, Lancaster LA1 4YG, UK; ^3^National Institute of Science and Technology for Molecular Entomology, 373 Carlos Chagas Filho Av., Center for Health Science, Building D, Basement, Room 5, Cidade Universitária, 21941-590 Rio de Janeiro, RJ, Brazil

## Abstract

*Lutzomyia longipalpis* is the main vector of visceral leishmaniasis (VL) in America. Physiological and molecular mechanisms of* Leishmania* infection in sand flies have been studied during the first gonotrophic cycle. There are few studies about these interactions during the second gonotrophic cycle mainly because of the difficulties maintaining sand flies through sequential feeds. Here we standardized conditions to perform the second blood feed efficiently, and our results show that oviposition is an essential factor for the success of multiple feeds. We evaluated the impact of the second blood meal on longevity, protein digestion, trypsin activity, and* Leishmania mexicana* development within* L. longipalpis* gut. Mortality of blood-fed females increases after second blood meal as compared to sugar-fed females. Trypsin activity was lower during the second gonotrophic cycle. However, no difference in protein intake was observed between blood meals. There was no difference in the population size of* Leishmania* in the gut after both blood meals. In this work, we presented an optimized protocol for obtaining sufficient numbers of sand fly females fed on a second blood meal, and we described some physiological and parasitological aspects of the second gonotrophic cycle which might influence the vectorial competence of sand flies.

## 1. Introduction

Visceral leishmaniasis (VL) is a severe chronic disease caused by protists belonging to the* Leishmania donovani* complex [1]. Currently, the estimated incidence of VL is 0.2 to 0.4 million cases per year with over 90% occurring in India, Bangladesh, Sudan, South Sudan, Ethiopia, and Brazil [2].

In the Americas, the phlebotomine sand fly* Lutzomyia longipalpis* is the main vector of VL [3]. Both male and female* L. longipalpis* adults, after emergence from pupae, feed on sugars from plants and aphids in a regular basis, but only adult females are hematophagous, feeding on a wide range of vertebrates as dogs, chickens, humans, and horses, among others [4]. After a saturating blood meal, the sand fly female starts its digestion, a process that takes an average of 3-4 days [5]. Female* L. longipalpis* are anautogenous insects, where egg development occurs only after a blood feed. The oviposition in this species starts 3 days after the blood meal and in general lasts for 6 days [6, 7]. A second blood feed is necessary to start a new cycle of digestion and oviposition [8].

During an infective blood meal, sand flies ingest infected macrophages that release amastigote forms of* Leishmania* parasites in the midgut. The amastigotes differentiate in the sand fly midgut to procyclic promastigotes, replicative forms which can survive and develop in the presence of microbiota [9, 10] and digestive enzymes [11–17]. After 2–4 days, procyclic forms develop into nectomonad forms, which escape from the peritrophic matrix by the posterior opening of this structure, attach to the intestinal epithelium, and migrate forward to the anterior thoracic midgut by chemotaxis/osmotaxis [13, 14, 18–24]. Nectomonad forms transform into leptomonad forms, multiplicative forms responsible for the parasites' second replicative cycle and secretion of promastigote secretory gel (PSG) [25]. Haptomonad forms colonize the stomodeal valve by attachment to the cuticular lining, blocking this structure physically, together with leptomonads and metacyclics that are embedded in the PSG [13, 14]. Metacyclics are the infective forms for the mammalian vertebrate and develop in the foregut or behind the stomodeal valve from leptomonad forms [25]. Their transmission to the vertebrate host occurs through a bite during a subsequent blood feed [14].

Trypsin is a key enzyme responsible for primary digestion of blood proteins in female sand flies [16].* Leishmania* parasites can modulate the activity of this enzyme during infection in the sand fly vector [11, 12, 15–17, 26]. Despite these studies, nothing is known about the regulation of digestion in infected flies during the second gonadotrophic cycle.

Elnaiem et al. [27] described the development of* Leishmania chagasi* parasites within* L. longipalpis* gut during the second gonadotrophic cycle. It was shown that the second blood feed is essential for the relatively rapid appearance of metacyclic forms and its fast migration to the vector proboscis, perhaps leading to an increase of vectorial competence in infected sand flies undergoing the second cycle. Similarly, Vivenes et al. [28] showed the rise of migration and colonization of* Leishmania mexicana* in* Lutzomyia evansi* esophagus after a second blood meal.

One crucial limitation for studying multiple gonotrophic cycles in sand flies is the difficulty in obtaining engorged females after a second blood feed. Rearing large colonies of sand flies in the laboratory is problematic due to high mortality after the first blood feed in contrast to breeding the commonly available mosquito species that can be easily maintained through multiple gonotrophic cycles. Previous work has evaluated the percentage of blood-fed females of* L. longipalpis* after first and second blood feeds and, in general, the number of fed females was much lower after the second blood feed than the first feed [8]. Stamper et al. [29] observed that* Phlebotomus duboscqi* sand flies that had laid eggs previously accepted a second blood meal more efficiently compared to insects that retained eggs. This result suggested that oviposition was an essential prerequisite for a successful second blood feed. Considering the critical role of the multiple blood feedings for* Leishmania* transmission, we aimed to investigate conditions that would promote efficient second blood feed in female* L. longipalpis *which ensured fly survival. We have developed a protocol to evaluate the impact of the second gonotrophic cycle on longevity, protein digestion, trypsin activity, and* Leishmania mexicana *development within* L. longipalpis* females.

## 2. Materials and Methods

### 2.1. Sand Flies Maintenance

*L. longipalpis*, originally from Jacobina (Bahia, Brazil), were kept at Lancaster University (United Kingdom) under standardized conditions of temperature (24 ± 2°C) and a photoperiod of 8 h of light/16 h of darkness. Adult insects (males and females) were fed ad libitum with 70% (w/v) autoclaved sucrose during all experiments.

Unless specified, sand flies received the first blood meal four days after emergence of adults. Second blood feeding was done seven days after first blood feeding (females were 11 days old). We chose these time points because in our conditions these are the minimal times to have successful first and second blood meals. Longer times would impair the time course analysis of blood digestion and infection due to high mortality rates (see below).

Sheep blood, with Alsever's anticoagulant (Cat. No. SB068), was purchased from TCS Biosciences (Buckingham, United Kingdom) and used in all experiments. Blood feeds were carried out via artificial apparatus (Hemotek, Discovery Workshops), with chicken skin membranes held at 37°C for 1 hour.

### 2.2. Percentage of Fed Females, Oviposition, and Counting of Developed Oocytes after Uninfected Blood Feeding

Females (*n* = 125) and males (*n* = 25) were added to cages with dimensions of 17 × 17 × 19 cm (height × width × length, resp.) for the first blood feed. After blood feeding, fed females were equally divided between 2 small cages (dimensions 11 × 11 × 14 cm, height × width × length, resp.), one of them containing a plastic oviposition pot inside of it (on the bottom of the cage). The pot (4.6 cm diameter × 4.3 cm height) included a 1 cm thick layer of plaster of Paris in the base, moistened with deionized water. These cages were maintained as described in sand fly maintenance section and after seven days, 25 females from each group were offered a second blood feed. The percentage of fed flies was estimated after visual inspection of blood engorgement. Immediately after the second blood feed, all females (engorged and nonengorged) were dissected in PBS or 0.9% w/v saline, and the number of oocytes within the body was counted. Because sand flies are anautogenous insects, we assume that the first blood meal does not interfere in the oocyte counting after the second blood feed. Females with small amounts of blood in the gut (partial blood feeding) were not counted as fed.

### 2.3. Longevity of Uninfected Females after First and Second Blood Meal

In an initial series of experiments, we compared sugar-fed and blood-fed females (first blood meal). For both groups, we used 4-day-old females (30–50 insects per group) in 17 × 17 × 19 cm cages.

In the second series of experiments, we monitored four different groups: sugar-fed only, females fed with blood 4 or 11 days after emergence, and females fed with blood twice at 4 (first blood meal) and 11 (second blood meal) days after emergence. We used 20–30 females for each group in 17 × 17 × 19 cm cages.

All groups were offered 70% sucrose ad libitum. Mortality was evaluated, and dead insects were removed from the cages daily.

### 2.4. *Leishmania mexicana* Infections

*L. mexicana* infections were performed using the axenic culture of amastigotes, strain M379. Amastigote forms of the parasite were cultured in Grace's Insect Medium supplemented with 20% of fetal bovine serum (FBS), 2% urine, 25 *μ*g/mL gentamycin sulfate, and BME vitamins and incubated at 32°C. Parasites with a maximum of 26 passages after isolation from the vertebrate host were used for sand fly infections. Parasite density was estimated using Neubauer chambers, and amastigotes were diluted in sheep blood (with Alsever's solution) to a final concentration of 2 × 10^6^ parasites/mL. Parasites were offered to females only during the first blood feed. Control insects were fed with uninfected blood, and unfed females were discarded. Fed females from control and infected groups were maintained in cages containing oviposition pots until the second blood meal. Seven days after the first blood feed, uninfected blood was offered to both groups. All blood feeds were carried out according to sand fly maintenance section.

### 2.5. Protein Quantification

Infected and control sand flies were dissected before and immediately after blood feed and 24, 48, 72, and 96 hours after first and second blood feed. Females were dissected in cold 0.9% (w/v) NaCl or phosphate buffered saline (PBS), and each gut (crop removed) was homogenized in 20 *μ*L of PBS. Samples were kept at −20°C until quantitation. Protein concentration was determined using bicinchoninic acid (BCA) and bovine serum albumin as standard [30]. Briefly, 10 *μ*L of homogenate was pipetted into PCR tubes and subsequently mixed with 40 *μ*L of BCA. Samples were incubated for 25 min at 80°C in a PCR thermocycler machine. After incubation, 40 *μ*L of incubated samples was read at 562 nm in a microplate reader with flat-bottom 96-well plates. All measurements were performed in the linear range of a standard curve (range 0–2 ug) assembled in the same plate (*R* = 0.993 ± 0.004, range = 0.273 ± 0.017 OD, background = 0.066 ± 0.009 OD, and resolution = 0.001 OD).

### 2.6. Trypsin Assays

Infected and control flies were dissected 24, 48, 72, and 96 hours after first and second blood feeds. Females were dissected in cold 0.9% (w/v) NaCl or PBS. Each gut (crop removed) was added to 20 *μ*L PBS and homogenized with a pellet pestle (Sigma-Aldrich Corp., Cat. No. Z359947). After that, 10 *μ*L of samples was diluted 5x in PBS and kept on ice. The substrate used in the experiments was N*α*-benzoyl-L-arginine 4-nitroanilide hydrochloride (L-BApNA, Sigma-Aldrich Corp.), stock concentration 9 mM diluted in dimethyl sulfoxide (DMSO).

Trypsin activity was assayed by a discontinuous assay. Briefly, 10 *μ*L of fresh homogenate was mixed in 70 *μ*L of Tris-HCl buffer (200 mM, pH 8.0) and 10 *μ*L of the substrate. Samples were incubated for 40 minutes at 30°C, and reactions were interrupted at different times (10, 20, 30, and 40 minutes) by adding 10 *μ*L acetic acid 50% (v/v). After the end of the assay, plates were read at 405 nm in a 96-well microplate reader (resolution 0.001 OD) for the measurement of released product. The amount of product was calculated from a standard curve of 4-nitroaniline (0–40 nmol; Cat. No. 185310, Sigma-Aldrich Corp.) assembled and read in the same conditions (range = 0.96 ± 0.05 OD,* R* = 0.996 ± 0.001, and background absorbance 0.0443 ± 0.0004). One unit of enzymatic activity (U) is the amount of enzyme which releases 1 *μ*mol product/min. Controls where the acid was added before the substrate (inactivated enzyme) were used as zero time readings. Controls without substrate or sample were incubated similarly.

### 2.7. Estimation of* Leishmania* Population Size in the Midgut

Infected females were dissected 3 or 6 days after first or second blood feed, and the guts were added to 20 *μ*L PBS containing paraformaldehyde (2% final concentration) in polypropylene tubes. Samples were gently homogenized with pestles in 1.5 mL polypropylene tubes, and the number of parasites was estimated with a Neubauer chamber using light microscopy.

### 2.8. Statistical Analysis

All statistical analysis was performed using GraphPad Prism 5.0 for Windows (San Diego, California, USA) and Shapiro-Wilk Normality Test (online version, http://sdittami.altervista.org/shapirotest/ShapiroTest.html) [31]. Unpaired Student's* t*-test was used for comparison of the normally distributed data, and Mann–Whitney test was used for comparison of the non-normally distributed data. Results are expressed as the group mean ± SEM Survival curves were compared using the log-rank Mantel-Cox test. Significance was considered when *p* < 0.05.

## 3. Results

### 3.1. Percentage of Fed Females, Oviposition, and Developed Oocytes after Uninfected First and Second Blood Meal

**Figure 1:**
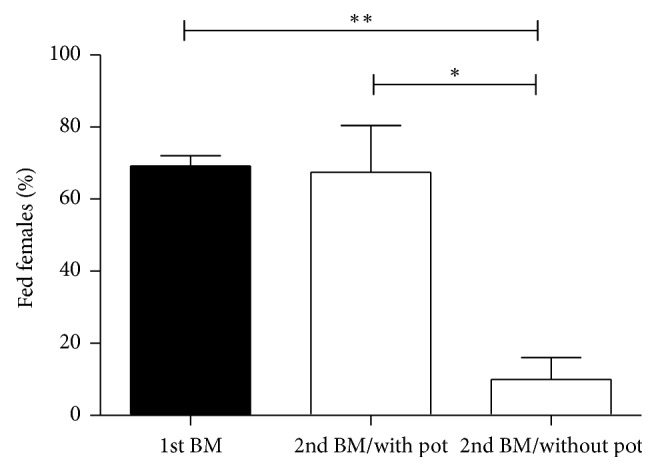
Effect of oviposition on the second blood feed of female* L. longipalpis*. Females were blood-fed and separated into cages in presence or absence of an oviposition pot. Seven days after the first blood meal, a second blood feed was offered. Percentage of blood-fed females was evaluated after the first and second blood meal. BM: blood meal, ^*∗*^*p* < 0.05; ^*∗∗*^*p* < 0.01 (unpaired* t*-test).

Preliminary experimentation and observation led to the hypothesis that females which oviposit after first blood feed would accept a second blood meal more easily than females which do not lay eggs after a first blood meal. To test this hypothesis, we fed* L. longipalpis* females with blood and separated the engorged ones in cages containing or not oviposition pots inside of them. In general, females laid a large number of eggs inside the oviposition pot after a first blood meal (data not shown), although few eggs could be observed outside the pot and very few on the net of cages. It was observed that 69 ± 3% of* L. longipalpis* females fed on the first blood meal. However, significantly lower percentage of females (10 ± 6%, *p* < 0.05) have accepted to feed on a second blood meal in the absence of oviposition pot inside rearing cages, compared to 67 ± 13% of females performing a second blood feed after oviposition. This percentage was similar (*p* > 0.05) to the percentage observed to undergo a first blood feed (Figure 1). The percentage of females with partial blood feed was very low, ranging from zero to 14% in all experiments. We did not observe significant differences comparing the cages with the oviposition pot to the ones without it in those percentages (data not shown).

**Figure 2:**
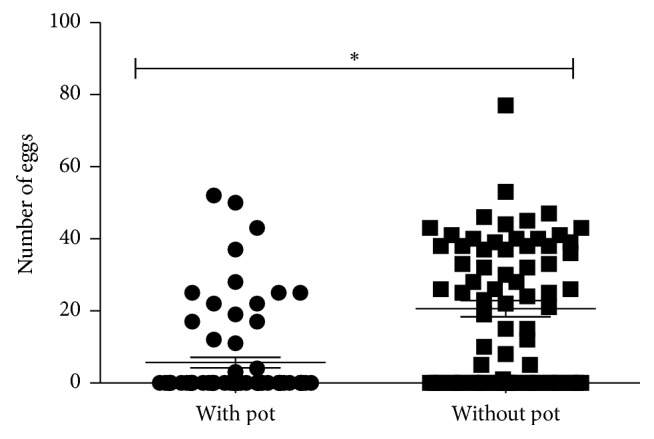
Number of retained oocytes in female* L. longipalpis*. Females were blood-fed and the engorged ones separated in cages in presence or absence of an oviposition pot. Seven days after the first blood feed, a second blood meal was offered. Immediately after the second blood feed, all females (fed and unfed) were dissected to estimate oocytes within the ovaries. ^*∗*^*p* < 0.05 (unpaired* t*-test).

To determine whether egg deposition was correlated with a successful second blood meal, all females (fed and unfed) previously kept in cages with or without oviposition pots were dissected immediately after the offering of a second blood meal, and the number of oocytes within the ovaries was recorded. The number of oocytes retained in females kept in cages without an oviposition pot was significantly higher (21 ± 2 oocytes/sand fly; *n* = 71 females) when compared to females maintained with the oviposition pot (6 ± 1 oocytes/female, *n* = 73 females, *p* < 0.05) (Figure 2). It is unlikely that the observation of oocytes retention was due to a short experimental duration, because most females in the group with pot finish oviposition in 5-6 days (data not shown).

### 3.2. Longevity of Uninfected Females after First and Second Blood Meal

**Figure 3:**
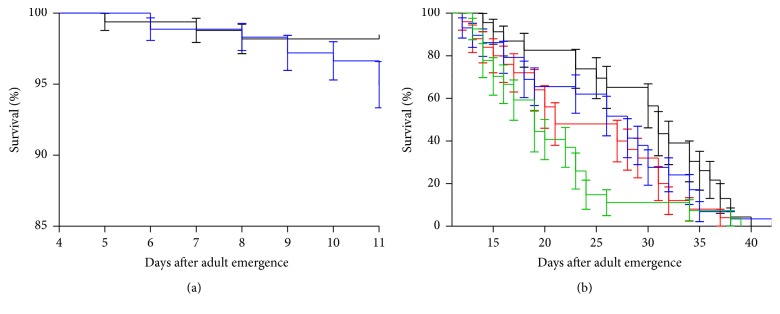
Survival curve of* L. longipalpis* females after (a) first and (b) second blood feed. Lines in (a) represent sugar-fed (black) and blood-fed (blue). Lines in (b) represent blood-fed once with four-day-old females (blue), blood-fed once with 11-day-old females (red), blood-fed twice (at 4 and 11 days after emergence, green), and sugar-fed only (black).

The effect of first and second blood feed on the survival of females was investigated after establishing optimal conditions for obtaining a significant proportion of engorged sand flies after the second blood meal. Blood feed had no significant effect on fly lifespan, when blood-fed females were compared to sugar-fed females until the seventh day after the first blood feed (*p* > 0.05, Figure 3(a)).

Nevertheless, under our experimental conditions, the second blood meal could be only offered to older flies, at least 11 days after emergence. An experiment was set up to investigate whether aging could exert some effect on the mortality observed after blood feed. The two populations of flies fed once, 4 or 11 days after emergence from pupae, had longevity curves that are similar to each other (*p* = 0.41, blue and red lines in Figure 3(b)). Interestingly, when we consider the full survival curve (until the death of the last fly), flies that blood-fed on day 4 after emergence showed a longevity curve similar to the sugar-fed ones (*p* = 0.19; blue and black lines in Figure 3(b)), and flies that blood-fed on day 11 after emergence showed a longevity curve different from the sugar-fed controls (*p* = 0.0289, red and black lines in Figure 3(b)).

Additionally, the effect of the second blood meal on 11-day-old flies that had fed on the first blood meal at four days after emergence was studied. Females that had two blood meals displayed significantly shorter longevity (green line in Figure 3(b)) than flies that fed only once on blood at 4 days after emergence (*p* = 0.0338, blue line in Figure 3(b)) and sugar-fed females (*p* = 0.0031, black line in Figure 3(b)). However, no difference was observed between females that blood-fed twice (green line in Figure 3(b)) and females that blood-fed only at 11 days after emergence (*p* = 0.31, red line in Figure 3(b)).

### 3.3. Protein Determination and Trypsin Assays of Uninfected and Infected Females after First and Second Blood Meals

**Figure 4:**
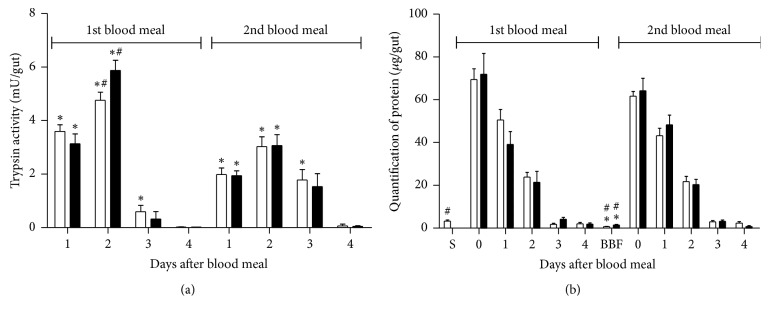
Trypsin activity (a) and quantification of protein (b) in control (white bar) and* Leishmania-*infected (black bar)* L. longipalpis* females. Females were infected with* L. mexicana* parasites in the first blood feed and fed with blood only in the second blood feed. Asterisk indicates statistical differences between first and second blood feeding and hashtag indicates statistical differences between infected and uninfected groups. S: sugar-fed; BBF: before blood feed.

The dynamics of the blood digestion after first and second blood feed in* L. longipalpis* females were evaluated through the measurement of trypsin activity and quantification of total protein in the sand fly gut. In general, trypsin activities after the second blood feed were lower when compared to activities after the first blood feed (Figure 4(a)). This difference was statistically significant 1–3 days after blood feed (*p* < 0.05) for uninfected females and 1 and 2 days for infected females. In both first and second blood meals, trypsin activities peaked at day 2, decreased at day 3, and were negligible 4 days after the feed (Figure 4(a)).

The effect of* Leishmania* infection on trypsin gut activity was evaluated by infecting* L. longipalpis* females with* Leishmania mexicana* via the first blood meal. Two additional sets of flies (infected and noninfected) were allowed to feed on a second noninfective blood meal and then trypsin activity was determined in both groups. The results are summarized in Figure 4(a). Gut trypsin activities of infected flies were similar to those observed in noninfected controls (*p* > 0.05). The only exception to this tendency was observed two days after the first blood feeding, when the activity of previously infected flies (6 ± 0.4 mU/gut) was significantly higher (*p* < 0.01) than the values obtained from noninfected controls (5 ± 0.3 mU/gut, Figure 4(a)).

Due to the alterations observed in gut trypsin activities of sand flies related to the second blood feed and* Leishmania* infection, we decided to investigate if these changes affected the overall protein digestion. We measured the total protein content in the gut after the first and second blood meals in noninfected and infected flies. Intestinal protein amounts after the second blood meal were similar to those observed after the first blood meal (*p* > 0.05, Figure 4(b)). The only difference recorded between first and second blood feeds was the protein content in the gut before blood feeds (Figure 4(b), *p* < 0.05). In general, the gut protein content in infected flies was similar to the values obtained from noninfected insects (Figure 4(b)).

### 3.4. Estimation of* Leishmania* Population Size in the Midgut

**Figure 5:**
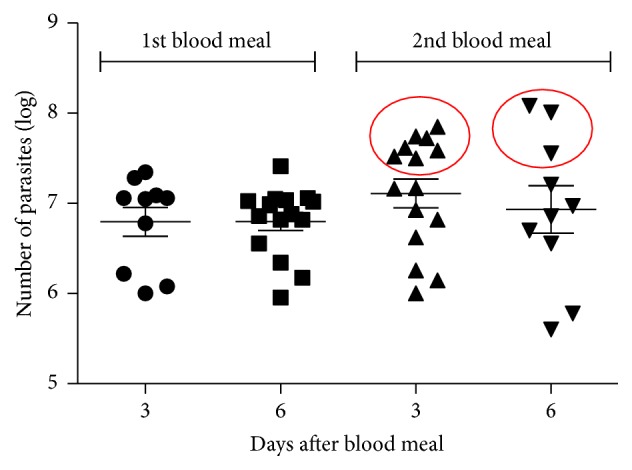
Population estimates of* Leishmania mexicana* promastigotes in the gut of* L. longipalpis* females after the first and second blood feed. Females were infected with parasites in the first blood feeding and fed only with blood in the second blood feeding. Red circle denotes heavily infected insects after the second blood feed.

**Figure 6:**
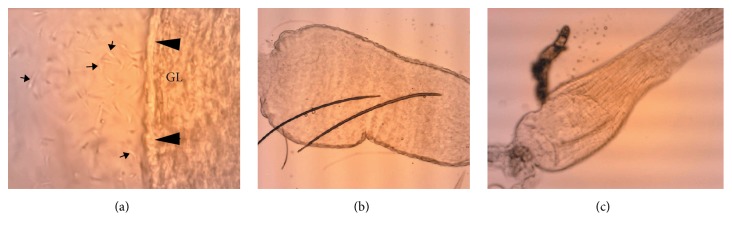
Light microscopy image of infected gut (a) and cardia after second blood feed in infected (b) and uninfected (c) sand flies. Females were infected with* L. mexicana* parasites in the first blood feeding and fed only with blood in the second blood feed. Note gut epithelium (arrowhead) and* Leishmania* parasites (arrows). GL: gut lumen. Magnification 100x.

The effect of the second blood feed on the development of the* L. mexicana* population was evaluated. Infections resulted in relatively high parasite populations in the midgut 3 and 6 days after the first blood meal. The second blood meal did not change average parasite counts (*p* > 0.05), but there was a trend towards an increase in the proportion of heavily infected insects. Although there was no statistical difference in average parasite density after the second blood meal, it was possible to observe a higher proportion of insects heavily infected after the second blood meal (Figure 5). To evaluate the migration of parasites forward towards the stomodeal valve (cardia region), we analyzed the morphology of this structure by light microscopy. Seven days after the second blood feeding the cardia of infected females was widely dilated when compared to uninfected females (Figure 6), suggesting a substantial population of parasites located within the structure.

## 4. Discussion

A major limitation to studies on multiple blood feeds by sand flies is to obtain sufficient females able to pass through a second gonotrophic cycle after a second blood meal. The method presented here was successful in routinely producing cohorts of* L. longipalpis* females taken through multiple blood feeds, allowing the study of* Leishmania* development through at least two gonotrophic cycles. It is also potentially of benefit when increasing the colony size by allowing for successive egg collections from the same batch of sand flies.

Several biochemical, physiological, and molecular factors have been studied about the interaction between* Leishmania* parasites and sand flies [9–12, 15–18, 20–29, 32–34]. However, all of these observations were obtained only with sand flies after the first blood feed.

Here, we show that facilitating conditions for oviposition after a first blood meal is a crucial step for a successful second blood feed. A similar observation was previously reported for* Phlebotomus duboscqi* females [29]. These conclusions suggest that, besides the blood meal being an essential step for maturation of ovaries, oviposition is necessary for multiple blood feed in* L. longipalpis.*

Despite the importance of blood feed for maturation of oocytes, our results showed that multiple blood meals could negatively affect the longevity of females. In general, hematophagous insects can ingest a large amount of blood, rich in proteins, mainly hemoglobin. Digestion of hemoglobin releases a significant amount of heme, a toxic molecule due to its capacity for generation of oxygen-reactive species or permeabilization of membranes [35]. Heme might be responsible for the increase in mortality of females that have taken more than one blood meal.

Blood meals possess large amounts of proteins, and hematophagous insects synthesize proteases for their digestion. Trypsin is the major insect gut protease, and it has been studied in sand flies [12, 15–17, 36] and almost all insect orders [37]. In mosquitoes, the production of trypsin in the midgut is divided into 2 phases after the blood feeding: initial and late trypsins, with minor and major activities, respectively [38]. In the sand fly* L. longipalpis*, two sequences of trypsin were previously described; however, only one trypsin transcript was induced after blood feeding [36].

Previous works have shown that* Leishmania* infection can modulate the activity of* L. longipalpis* trypsin. This enzyme was reduced in the presence of* Leishmania* parasites at certain time points [15–17]. Interestingly, our data suggest that trypsin activity is lower after second gonadotrophic cycle, regardless of infection, and that* L. mexicana* infections do not modulate the protease activities of* L. longipalpis*. The decrease of trypsin activity in the first and second day after second blood feeding, when compared to the noninfected first meal, ranged from 36 to 46%, being higher than the inhibitions previously described and correlated to parasite infections (mostly ranging from 16 to 37%, with an isolated observation of 60% inhibition in minor activities). This trypsin modulation after second blood meal might result in a favorable environment for previous infections and might represent a physiological condition inside the vector that is exploited by parasites.

In consonance to our data, Secundino et al. [39] also concluded that* L. major* infection does not modulate trypsin activity in* Phlebotomus duboscqi*. Nevertheless, it is important to consider all the variations between the reports on this subject. Relevant parameters as species of* Leishmania* and sand flies, gut microbiota, concentration and form of* Leishmania* parasites, blood source, and protocol for trypsin assays vary considerably. Another important factor, which is not even described in the literature and which might affect trypsin activities, is the anticoagulant added to the blood for artificial feedings (our was Alsever's solution). Several differences between some recent works about the effects of* Leishmania* on* L. longipalpis* trypsin are summarized in Table 1 and, for us, it is quite clear that research on this topic would greatly benefit from more standardization, at least for enzymatic assays. It is important to remember that for any invertebrate enzyme recommended parameters are 30°C in conditions where product release is proportional to protein amount and time [37].

It has been postulated that* Leishmania *infections pose difficulties for sand fly multiple blood feeds [19, 33, 40]. However, we have not detected differences regarding the ingestion of blood when comparing infected and uninfected females in the second blood feeding. In this respect, it is important to mention that our blood feeds were done through an artificial apparatus, and sand flies were not exposed to the normal blood homeostatic response from the vertebrate host and the associated interplay of salivary proteins and anticoagulants. Besides that, only a fraction of our infected flies were heavily infected, and the second blood feed was offered after only 7 days of infection. Possibly at later stages of infection the parasites may exert more detrimental effects in blood ingestion.

There were no gross differences in density of parasites in the gut of females within both gonadotrophic cycles, at 3 or 6 days after blood feeding. However, we observed a considerable variation of the parasitic load in the same experimental group when comparing individual sand flies. The same kind of variation of infection in* L. longipalpis* females infected with* L. mexicana* after the second blood feed was reported by Rogers et al. [40].

An alternative explanation for this variation in parasite numbers might be that in all of our experiments the blood feeding was offered without heat-inactivation of the blood, as we initially infected the sand flies with the amastigote forms of the parasite. This ingested blood might have resulted in parasite killing by the plasma from the second blood meal or additionally by some salivary, gut, or immune response of sand fly females due to the exposure of vertebrate blood compounds. However, inhibition of complement classical pathway of the vertebrate host by saliva from* L. longipalpis* has been described previously [41]. Additionally, sand fly digestive enzymes (including trypsins), which may have detrimental effects on the parasites, probably vary among individuals, being another putative important source of fluctuation in the parasite density in the population.

An increase in the size of the stomodeal valve (cardia region) in infected females was observed in the present study. Similarly, Rogers et al. compared the diameters of the cardia and thoracic midgut of* L. longipalpis *females uninfected and infected with* L. mexicana* and showed that these regions increased 1.5- and 1.7-fold in infected flies, respectively [40].

In conclusion, the effect of multiple blood feeds on the development of* Leishmania* in its vector is an essential parameter to understand the natural transmission of these parasites, longevity of* Leishmania*-infected sand flies, and ultimately vector-borne aspects of the epidemiology of leishmaniasis.

## 5. Conclusions

We have standardized a method to increase the number of engorged females after a second blood feeding, which will facilitate future work in this research area. Additionally, we have shown that midgut trypsin activity changes according to the gonadotrophic cycle, which might ultimately influence the vectorial competence of sand flies and transmission of disease.

## Figures and Tables

**Table 1:** Comparison of conditions and protocols used to study the effect of *Leishmania* infections on *Lutzomyia longipalpis* midgut trypsin.

Parameter	Santos et al. 2014	Telleria et al. 2010	Sant'anna et al. 2009	This work
Parasite species	*L. infantum*	*L. (infantum) chagasi*	*L. mexicana*	*L. mexicana*
Parasite form used in infections	Amastigote	Promastigote	Amastigote	Amastigote
Parasite concentration in infection	Natural infection	10^7^ parasites/mL	10^6^ parasites/mL	10^6^ parasites/mL
Blood source	Dog	Hamster	Rabbit	Sheep
Trypsin substrate	L-BA*p*NA (0.5 mM)	DL-BA*p*NA (2 mM)	BA*p*NA (0.88 mM)	L-BA*p*NA (1 mM)
Assay buffer (pH, final concentration)	Tris/HCl (pH 7.5, 50 mM)	Tris/HCl (pH 8.0, 10 mM)	Tris (pH 8.5, 88 mM)	Tris/HCl (pH 8.0, 155 mM)
Assay temperature (°C)	30	25	Ambient temperature (~22)	30
Incubation time (minutes)	30	60	5	40
Activity presented in	U^a^/midgut	mU/midgut	U/mg protein	mU/gut

^a^One unit of enzymatic activity (U) is the amount of enzyme which releases 1 *µ*mol product/min.

## Abbreviations

VL: Visceral leishmaniasis
PPG: Proteophosphoglycan
FBS: Fetal bovine serum
PBS: Phosphate buffered saline
BCA: Bicinchoninic acid
PCR: Polymerase chain reaction
L-BApNA: N*α*-Benzoyl-L-arginine 4-nitroanilide hydrochloride
DMSO: Dimethyl sulfoxide
fPPG: Filamentous proteophosphoglycan.
